# Pre-Concentration Freezing Alters the Composition of Mesenchymal Stem/Stromal Cell-Conditioned Medium

**DOI:** 10.3390/biology14020181

**Published:** 2025-02-10

**Authors:** Francesca Cadelano, Chiara Giannasi, Alice Gualerzi, Martina Gerli, Stefania Niada, Elena Della Morte, Anna Teresa Brini

**Affiliations:** 1 Department of Biomedical, Surgical and Dental Sciences, University of Milan, 20100 Milan, Italy; francesca.cadelano@unimi.it (F.C.); anna.brini@unimi.it (A.T.B.); 2 Laboratory of Biotechnological Applications, IRCCS Istituto Ortopedico Galeazzi, 20157 Milan, Italy; stefania.niada@grupposandonato.it (S.N.); elena.dellamorte@grupposandonato.it (E.D.M.); 3 IRCCS Fondazione Don Gnocchi Onlus, 20148 Milan, Italy; agualerzi@dongnocchi.it (A.G.); mgerli@dongnocchi.it (M.G.)

**Keywords:** stem cell-based therapy, stem cell conditioned medium, stem cell-derived secretome, biologics production, protocol standardization, cell-free biotherapeutics

## Abstract

Batch-to-batch reproducibility and robust quality assessment are crucial for producing cell-free biologics, such as conditioned medium (CM) derived from mesenchymal stem/stromal cells (MSCs). This study investigated the effects of freezing CM at −80 °C prior to concentration, a step that could occur in large scale pipelines, compared to freshly processed CM. Quality assessment included total protein quantification; extracellular vesicle evaluation using nanoparticle tracking analysis (NTA), transmission electron microscopy (TEM), and cytofluorimetry; and biochemical analysis using Raman spectroscopy. The freezing process resulted in a 34% reduction in total protein content, as confirmed for selected bioactive mediators, and significant depletion of specific particle types, particularly larger ones. Interestingly, the total particle concentration and polydispersity remained stable. Alterations in Raman spectra highlighted changes in protein, lipid, and nucleic acid content. These findings demonstrate that even routine steps like freezing can alter CM composition, likely due to temperature-induced structural changes in biological molecules. Careful consideration of pre- and intra-processing handling temperatures is critical for preserving the integrity of CM and ensuring consistent quality. This study emphasizes the importance of refining manufacturing protocols in the production of cell-free biologics.

## Simple Summary

Conditioned medium (CM) is a complex mixture of proteins, lipids, nucleic acids, and extracellular vesicles naturally produced by cells. It holds promising potential for medical applications, but maintaining consistent quality across batches is essential to ensure reliable safety and efficacy. This study examined the impact of freezing freshly collected CM at −80 °C, a common step in larger production pipelines, compared to processing it immediately after collection. Results showed that freezing caused a 34% reduction in total protein content, a decrease in the proportion of larger vesicles, and changes in spectral and compositional fingerprints. These findings underscore the significant effects that routine manufacturing steps, such as freezing, can have on CM composition. The sensitivity of biological molecules to temperature shifts, which can disrupt their structural organization, likely accounts for these changes. Addressing such variability is crucial for developing consistent and effective CM-based applications.

## 1. Introduction

The production of conditioned medium (CM) from various cell types, including mesenchymal stem/stromal cells (MSCs), has garnered significant interest as a promising bioproduct, harnessing the therapeutic potential of cell-secreted factors for a wide range of biomedical applications [1]. These include therapies for musculoskeletal disorders and inflammatory conditions such as arthritis or acute colitis, as well as applications in soft tissue regeneration and immunomodulation [2,3,4]. CM is enriched with soluble factors and vesicle-embedded components, a complexity that enables its use across various medical fields such as orthopedics, where CM is spearheading orthobiologics research in recent years. However, CM complexity also hinders its standardization, making reproducibility and stability across different production batches a significant challenge [5]. Due to the diverse composition of CM as a cell-free product, conventional methods for quantifying an active ingredient, as well as standard metrics like cell viability, are inapplicable to certify its quality. Moreover, CM is not identifiable as an advanced therapeutic medicinal product, for which the European Medicines Agency has approved guidelines [6,7]. Therefore, to expedite CM’s availability to a wider audience, a comprehensive panel of alternative and case-specific parameters should be considered to ensure product consistency. Given that the bioactive profile of CM is determined by both its soluble and vesicular components, characterizing extracellular vesicles (EVs) using established techniques such as nanoparticle tracking analysis (NTA), cytofluorimetry, and transmission electron microscopy (TEM) presents a viable approach. Furthermore, assessing the concentrations of major groups of proteins, lipids, and other biologically active molecules is essential for a robust quality assessment [8]. In addition to compositional characterization, the stability and shelf-life of CM are critical factors for its successful translation to clinical use and downstream applications. Lyophilization or cold storage, typically at −80 °C, are widely adopted methods for preserving CM for longer periods, with or without the addition of cryoprotective agents [9]. If the impact of multiple cycles of freezing and thawing is demonstrated to affect biomolecule activity [10], the impact of pre-processing cold storage on freshly collected CM has not been thoroughly examined. Often, interim storage of CM is necessary due to batch processing requirements or logistical constraints, but this step could introduce changes in CM composition and its bioactive profile. These freezing-induced alterations could have significant implications for temperature-sensitive components [9,11,12] and the reproducibility of CM-based therapeutics. This study aims to investigate the potential consequences of low-temperature exposure on CM composition, specifically when freezing occurs prior to CM concentration. A freezing–thawing step was incorporated to simulate scenarios where pausing or halting the production pipeline might be necessary or convenient. Such scenarios include the need for larger product quantities, necessitating the pooling of multiple production cycles, or the implementation of energy-intensive or time-consuming steps, such as freeze-drying. In these cases, the process can be optimized by performing the critical step concurrently across multiple batches. Our findings underscore the importance of carefully considering the conditions and timing of cold storage during the production process, as these factors may inadvertently affect the final product’s quality and therapeutic potential [13].

## 2. Materials and Methods

### 2.1. Conditioned Media Production

Cell culture supernatants were collected from 72-h, starved confluent adipose–derived stem/stromal cells (ASCs). Briefly, human ASCs from subcutaneous fat waste tissue collected after surgery at IRCCS Ospedale Galeazzi—Sant’Ambrogio with patient informed consent and ethical approval (TENET–IRCCS Ospedale San Raffaele Ethics Committee, approval number 38/int/2022) were isolated using 0.75 mg/mL collagenase type I (Worthington Biochemical Corporation, Lakewood, NJ, USA). The patients’ age and type of intervention are described in Table 1.

Cells were cultured in high-glucose DMEM supplemented with 10% fetal bovine serum (FBS HyClone, Euroclone, Pero, Italy), 2 mM glutamine, 50 µg/mL streptomycin, and 50 U/mL penicillin. Only cells between III to VII passage were used. Once confluent, they were shifted to starving medium (0% FBS) and cultured for 3 days. Afterwards, cells were detached and counted, and conditioned medium was collected. Further CM manipulation was done keeping the product on ice. After a first centrifugation to remove cell debris (10′, 4 °C, 2500× *g*), it was either frozen at −80 °C (thawed CM, T-CM) or freshly concentrated (fresh CM, F-CM). The freezing of starting volumes of 20 mL took approximately 20 min, after which the samples were stored at least overnight at −80 °C. Thawing was performed at 4 °C and required approximately 4 h. Amicon Millipore (Millipore, Burlington, MA, USA) filtering devices with 3 kDa cut-off porous membranes were used to concentrate the CM, for 90 min at 4 °C and 4000 g. Retrieved volume was quantified and stored at −80 °C for downstream analysis. Additionally, a pool for each protocol was also created to minimize variability.

### 2.2. Protein Quantification

Total protein content was evaluated using the Bradford assay (Bio-Rad, Milan, Italy) following a standard protocol. Observed OD values of samples were interpolated with standard curves to infer μg of protein and normalized to 10^6^ donor ASCs.

### 2.3. Luminex Discovery Assay

The levels of selected bioactive molecules in three pairs of F- and T-CM samples were measured using the Human Premixed Multi-Analyte Kit LXSAHM (R&D Systems, Minneapolis, MN, USA) according to the manufacturer’s protocol. This kit was designed to detect and quantify CCL2, CCL3, IL4, IL8, HGF, M-CSF, OPG, and VEGFA. Following appropriate dilution (1:2 and 1:500), samples were analyzed with the Bio-Plex Multiplex System (Bio-Rad, Milan, Italy). Data were processed using MAGPIX PONENT 4.2 software (Luminex Corporation, Austin, TX, USA).

### 2.4. Nanoparticle Tracking Analysis

The concentration and size distribution of CM particles were evaluated using nanoparticle tracking analysis (NTA). Volumes corresponding to 3–4 × 10^5^ cells were resuspended in 700 μL of triple-filtered PBS (with 0.22 μm sterile filters). The NanoSight (Malvern PANalytical, Salisbury, UK) set-up parameters comprised 3 × 60 s measurements and a 30 infusion rate. The quality of the analysis was confirmed based on three canonical values: 10^6^ to 4 × 10^9^ concentration of particles per mL, 20–120 particles per frame, and >20% valid events in total.

### 2.5. Cytofluorimetry for EV Characterization

For cytofluorimetric analysis, F- and T-CM pools were diluted 1:10 in 0.22 μm triple-filtered PBS and stained with the green fluorescent dye carboxyfluorescein diacetate succinimidyl ester (CFSE, Biotium Fremont, CA, USA). All data were obtained using a CytoFLEX flow cytometer (Beckman Coulter, Brea, CA, USA). Instrument calibration was set using a mix of Megamix-Plus SSC and FSC (Biocytex, Marseille, France), a reference bead mixture composed of FITC fluorescent spheres of known dimensions (100 nm, 160 nm, 200 nm, 240 nm, 300 nm, 500 nm, and 900 nm). Briefly, diluted CM aliquots were incubated with 1 μM CFSE for 1 h at 37 °C and then analyzed to confirm successful staining. CFSE-positive (CFSE+) CM samples were analyzed in Violet SSC–H and FITC–H channels to delimit the areas of interest, accordingly to the coordinates provided by the standardization beads. CFSE+ samples were then subdivided and incubated for 30 min at 4 °C in the dark with APC-conjugated antibodies αCD9, αCD63, and αCD81 (BioLegend, San Diego, CA, USA, dilution 1:20) then diluted in triple-filtered PBS to a final volume of 300 µL. Samples were acquired for 150 s at a medium flow rate. DMEM-diluted antibodies and unlabeled samples were used as appropriate controls.

### 2.6. Composition Analysis Using Raman Spectroscopy

Representative pairs of F-CM and T-CM samples were acquired using Raman spectroscopy by the Laboratory of Nanomedicine and Clinical Biophotonics (LABION) of IRCCS Fondazione Don Gnocchi. This technique investigates the compositional spectra of the samples, returning information on the content of lipids, proteins, and other molecules. Briefly, samples were diluted 1:10 in sterile saline solution, deposited on a calcium fluoride slide, and acquired following a previously optimized protocol for EVs and CM analysis [14,15] with a LabRAM Aramis Raman microspectroscope (Horiba Jobin Yvon S.A.S., Lille, France) equipped with a 532 nm laser line. Polynomial baseline correction and unit vector normalization were performed using Labspec 6 software (Horiba).

### 2.7. Assessment of Extracellular Vesicle Morphology with Transmission Electron Microscopy (TEM)

Aliquots of pooled F-CM and T-CM were diluted 1:5 and then analyzed using TEM at the UNITECH facility of University of Milan.

### 2.8. Analysis of Anticatabolic Action and Chemotactic Properties

The functionality of F-CM and T-CM was evaluated using two distinct models: an ex vivo cartilage explant model and an in vitro transwell migration assay with the monocytic cell line THP-1. To minimize inter-donor variability, pooled F-CM and T-CM samples were prepared and utilized for the functional assays. In the cartilage explant model, explants were harvested and treated following the protocol described by Cadelano et al. [16]. Experimental groups included untreated samples (control, CTR), cytokine-stimulated explants (10 ng/mL TNFα + 1 ng/mL IL-1β, TNF + IL), and cytokine-stimulated explants treated with F-CM or T-CM corresponding to 2.5 × 10^5^ ASCs (TNF + IL + F-CM and TNF + IL + T-CM, respectively). After 24 h, the activity of matrix metalloproteinases (MMPs), a catabolic marker of inflamed cartilage, was measured using the SensoLyte 520 Generic MMP Assay Kit (AnaSpec Inc., Fremont, CA, USA). Results were expressed as arbitrary fluorescent units (AFUs). Chemotactic properties were evaluated using the protocol implemented in Giannasi et al., employing 8 μm pore size transwells [17]. In this assay, 5 × 10^5^ calcein-stained THP-1 cells (ATCC TIB-202, Manassas, VA, USA) were seeded onto the inserts. The lower chambers contained serum-free RPMI alone (negative control, CTR−) or supplemented with F-CM or T-CM derived from 5 × 10^5^ ASCs. A positive control (CTR+) consisting of RPMI with 10% FBS was also included. After 6 days, the fluorescence of cells that migrated to the lower chamber was measured using a Wallac Victor II microplate reader (Perkin Elmer, Milan, Italy) and expressed as AFUs.

### 2.9. Statistical Analysis

The data were analyzed using GraphPad Prism (Version 10.4.1). All quantitative data were normalized to 10^6^ donor ASCs. Paired Student’s *t* tests were performed to assess significant differences between the two groups, upon evaluation of data distribution. For the functional assays, one-way ANOVA (analysis of variance) was performed. Only *p* values < 0.05 were considered significant (<0.05 *; <0.01 **; <0.001 ***). Raman data were analyzed using descriptive and multivariate statistics with Origin2023b (OriginLab, Northampton, MA, USA). The mean spectra of F-CM and T-CM samples were obtained. Principal component analysis (PCA) was performed followed by linear discriminant analysis (LDA) to verify the spectral differences between the samples. The nonparametric Mann–Whitney test was used to assess significant differences between the canonical scores of two groups. The protein to lipid ratio was calculated as previously described [14]. Briefly, the area under the curve in the spectral interval corresponding to amide I (AUC 1600–1690) and amide III (AUC 1200–1300) and in the spectral range with the main contribution of lipids (AUC 2750–3040) were calculated using the integration function in OriginLab. Then, the ratio between both amide I and amide III and lipid scores was calculated. Additionally, the area under the curve in the spectral range corresponding to nucleic acids (720–800) was obtained using the integration function in OriginLab. Subsequently, the ratio between nucleic acid and amide I and amide III scores was calculated.

## 3. Results

### 3.1. Protein and Particle Content Were Altered in T-CM

**Figure 1:**
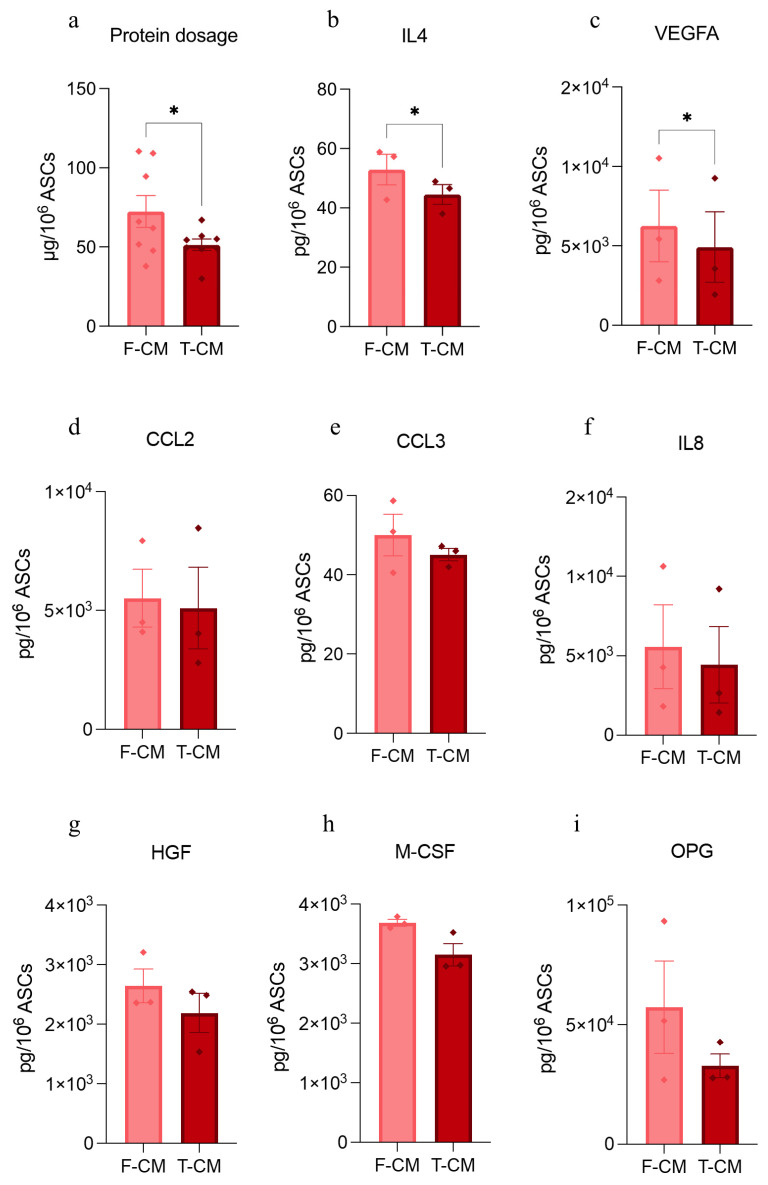
**Protein content and specific molecules quantification:** (**a**) Bradford protein quantification normalized to 10^6^ donor ASCs. Results are derived from *n* = 8 independent experiments and are represented as mean ± SEM. (**b**–**i**) Bar plots representing levels of eight different effectors in F-CM and T-CM, expressed as pg/10^6^ ASCs. Data are derived from *n* = 3 samples and are represented as mean ± SEM. * *p* < 0.05.

As part of the quality control, the total protein quantification was performed on all pairs of F-CM and T-CM to evidence possible differences. Additionally, several key molecules were also quantified in both products. The results showed a 34% reduction of total protein content (Figure 1a) in the T-CM compared to the F-CM. A significant reduction in IL4 and VEGFA (Figure 1b,c) was observed in T-CM compared to F-CM, while CCL2, CCL3, IL-8, HGF, M-CSF, and OPG exhibited a downward trend without reaching statistical significance (Figure 1d–i).

**Figure 2:**
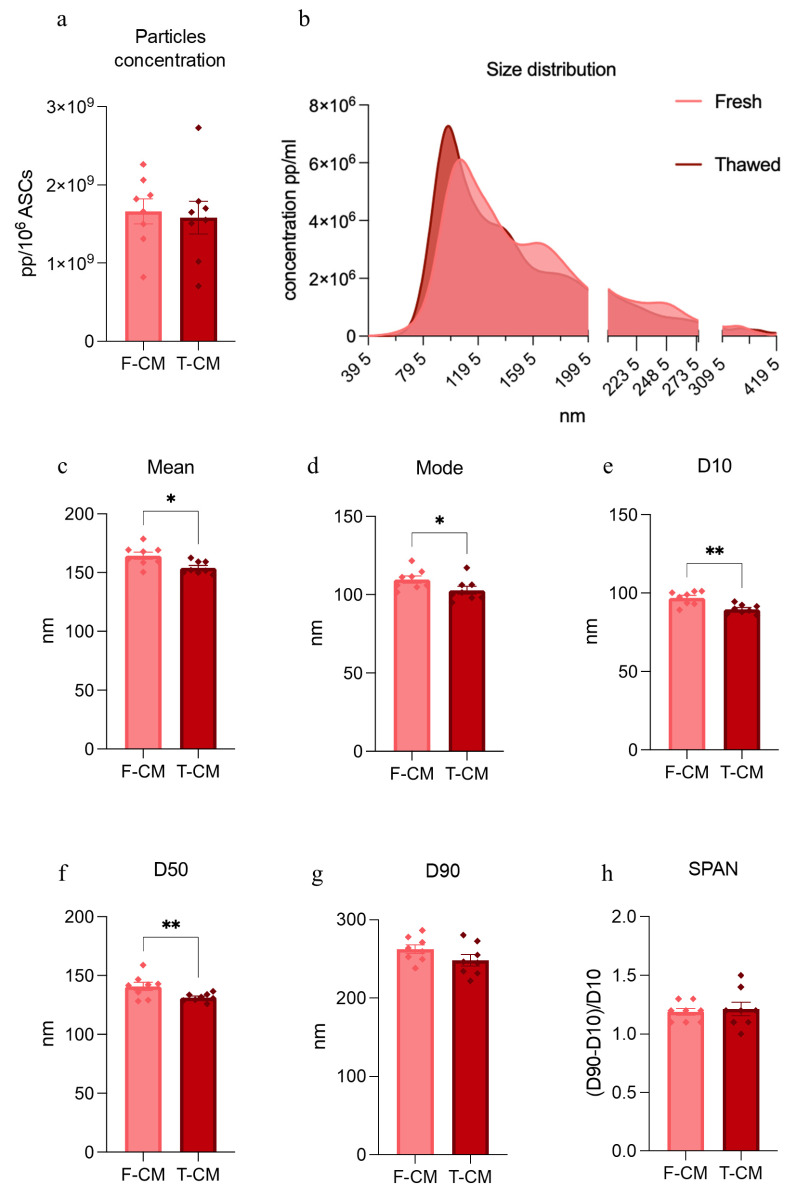
**Particles concentration and distribution metrics in F-CM and T-CM**: (**a**) Total particle concentration measured using a nanoparticle tracking assay (NTA) and normalized to 10^6^ ASCs. (**b**) NTA mean curves that show particle (pp) distribution and major variations between F-CM and T-CM. (**c**,**d**) Bar plots representing the numerical values obtained from NTA (mean–mode). (**e**–**g**) Bar plots representing D10-D50-D90 diameters obtained with NTA. (**h**) Span index was calculated as (D90–D10)/D50. A span greater than 1 indicates a heterogenous sample. Data are derived from eight independent experiments (*n* = 8) and are represented as mean ± SEM. * *p* < 0.05, ** *p* < 0.01.

The NTA of our samples reported that the concentrations were unaltered between F-CM and T-CM (Figure 2a); however, they showed two different curves of particle distribution (Figure 2b). In detail, the distribution curves, obtained by the combination of particles with a specific bin diameter, confirmed a shift in the T-CM toward smaller bins, compared to F-CM, with the decrement of some peaks (e.g., regions around 150–200 nm and 220–280 nm). The NTA also provides statistical information on the particles size distribution. We observed significant decrements of mean (−6.4%), mode (−6.6%), D10 (−7.4%), and D50 (−7.1%) bin diameters in T-CM samples (Figure 2c–f), suggesting that smaller particles are now present in the thawed samples. D90 diameters did not change between the two groups (Figure 2g). From these data, it is possible to extrapolate an indication of the dispersity of the sample, such as the span calculated as (D90–D10)/D50, which is generally around 1 for highly heterogenous and polydisperse samples, such as CM [18]. Notably, the span indexes showed that the two CM do not differ in distribution width (Figure 2h).

### 3.2. TEM and Cytofluorimetric Analysis of Vesicular Components Confirmed EV Quality

**Figure 3:**
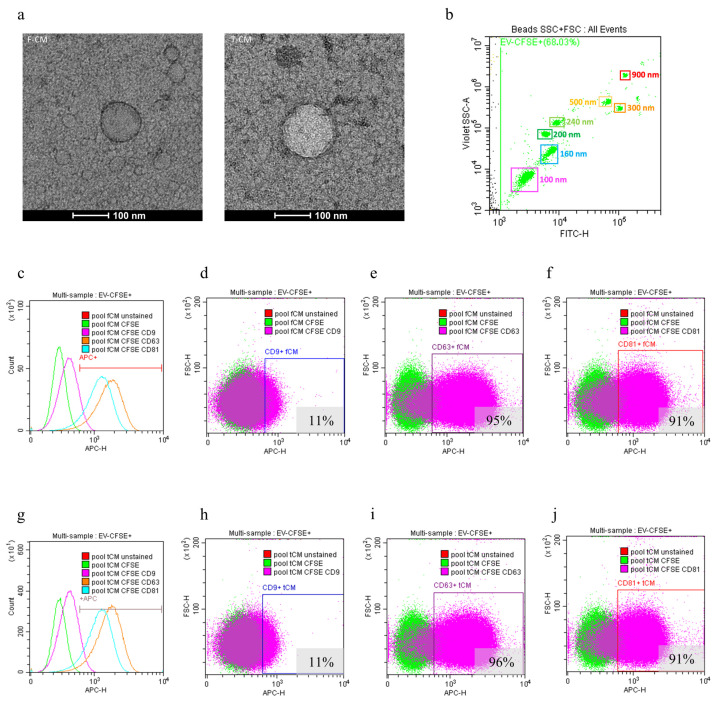
**EV characterization using pooled F-CM and T-CM:** (**a**) Representative images of EVs in F-CM and T-CM. Diluted 1:5 samples were prepared for TEM analysis using negative staining. Images were acquired at 57k× magnification. (**b**) Calibration for dimensional gating and debris exclusion was made using FITC+ beads with known sizes (corresponding diameters of 100, 160, 200, 240, 300, 500, and 900 nm). (**c**) F-CM CFSE+ vesicles that showed positivity for CD9 (**d**), CD63 (**e**), and CD81 (**f**). (**g**–**j**) T-CM following the same pattern as described above.

As a proof of concept of the presence of intact EVs, and to assess their morphology, TEM analysis was performed on F-CM and T-CM. Acquired images confirmed the presence of defined EVs in both preparations (Figure 3a). The cytofluorimetric evaluation of vesicles is essential to compensate the limit of NTA and also to verify proper EV quality by assessing their surface marker expression, such as CD9, CD81, and CD63. Our samples contained CFSE+ EVs in dimensional ranges consistent with known EVs dimensions (Figure 3b). They also showed positivity for the canonical vesicular markers, with F-CM showing 11% CD9+, 95% CD63+, and 91% CD81+ (Figure 3c–f). T-CM showed almost equal characteristics with 11% CD9+, 96% CD63+, and 91% CD81+ (Figure 3g–j). The percentages were totally comparable between F-CM and T-CM; therefore, the freezing process does not appear to influence the quality of the vesicles.

### 3.3. Raman Spectroscopy Highlights Biochemical Differences in the Two Differently Processed CM

**Figure 4:**
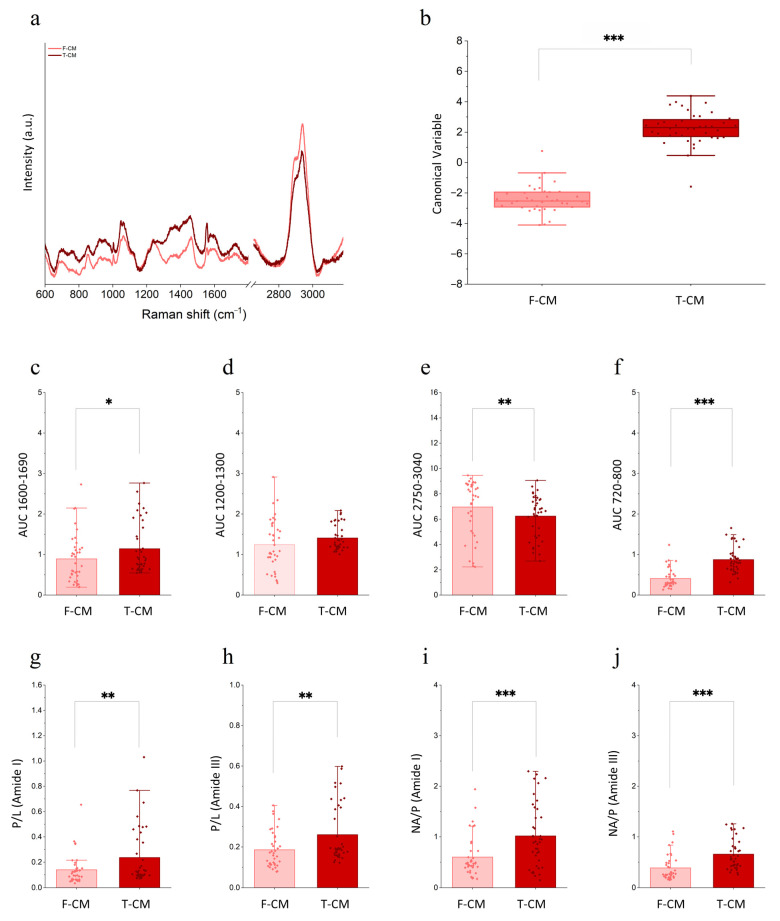
**Biochemical characterization of F–CM and T–CM using Raman spectroscopy:** (**a**) Average Raman spectra of *n* = 4 independently analyzed F-CM and T-CM. Average spectra are obtained from a minimum of 10 spectra per sample. (**b**) Box plot of the canonical variable scores obtained after PCA and LDA. (**c**–**f**) Bar plot of the spectral area under the curve (AUC) in the amide I range (1600–1690 cm^−1^), the amide III range (1200–1300 cm^−1^), the lipid associated range (2750–3040 cm^−1^), and the nucleic acids range (720–800 cm^−1^). (**g**,**h**) Bar plot of the protein to lipid ratio (P/L) obtained for F-CM and T–CM from the AUC of amide I and amide III divided based on lipid range. (**i**,**j**) Bar plots of the ratios of nucleic acids to both amide I and amide III, derived from the respective AUC ratio. * *p* < 0.05, ** *p* < 0.01, and *** *p* < 0.001.

The comprehensive biochemical evaluation of CM can be performed using Raman spectroscopy. From the Raman spectra, it is possible to appreciate differences between F-CM and T-CM, particularly in the 1200–1600 cm^−1^ and 2800–3000 cm^−1^ ranges (Figure 4a, and Supplementary Figure S1). These spectral regions are associated with the vibrational profiles of multiple molecules, including protein secondary structures (i.e., amide I around 1650 cm^−1^ and amide III around 1240–1300 cm^−1^) and lipidic bonds, making specific molecular attribution challenging. Nonetheless, the variations in the intensity in the 1200–1600 cm^−1^ range, which includes the amide I and amide III bands, might indicate that proteins are affected by the pre-processing freezing. The multivariate statistical analysis applied to all the acquired spectra demonstrated two distinguished profiles for F-CM and T-CM (Figure 4b), suggesting that we obtain two different final products, in terms of biochemical composition, derived from starting samples that differ only in pre-processing handling. The integration of the spectra highlighted the main differences in the amide regions AUC 1600–1690 (Figure 4c) and AUC 1200–1300 (Figure 4d), as well as in those bands corresponding to lipids (AUC 2750–3040; Figure 4e) and nucleic acids (AUC 720–800; Figure 4f). Trying to further understand the source of the observed chemical variations, the spectral protein to lipid (P/L) ratio as well as nucleic acids to protein (NA/P) ratio were calculated as described in Section 2.9 [14]. Interestingly, Raman data show a significant variation in protein and lipid content in the T-CM samples compared to F-CM, resulting in an overall increase in the spectral P/L ratio (Figure 4g,h). Moreover, the evaluation of the NA/P ratio suggests an increased contribution of nucleic acids in the spectral fingerprint of T-CM compared to F-CM (Figure 4i,j) that is expected to correspond to a higher concentration of free or particle-encapsulated nucleic acids (miRNAs, long non-coding RNAs, etc.). It has to be noted that the increased spectral contribution of nucleic acids in T-CM might be also related to the release of nucleic acids from particles damaged by the freezing–thawing cycles. Indeed, once small nucleic acids are encapsulated within lipid particles like EVs, their spectral contribution is often hidden by the strong lipid and protein signals.

### 3.4. T-CM Exhibits Anticatabolic and Chemotactic Potential Comparable to F-CM

**Figure 5:**
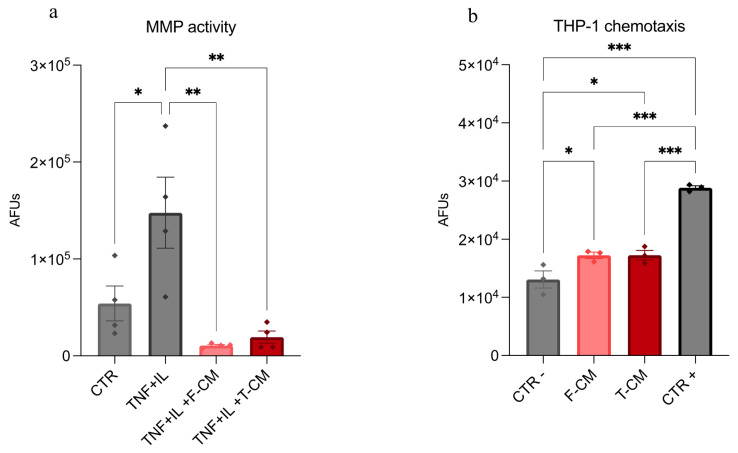
**Anticatabolic and chemotactic effects of F-CM and T-CM**: (**a**) Bar plots representing generic MMP activity in cartilage explants supernatants. MMP activity is expressed as AFUs. Data are derived from *n* = 4 independent experiments and are represented as mean ± SEM. (**b**) Bar plots representing the intensity of fluorescence, expressed in AFUs, detected in the bottom chamber of the transwell model after 6 days. AFUs directly correlate to the presence of recruited THP-1. Data are derived from *n* = 3 independent experiments and are represented as mean ± SEM. * *p* < 0.5, ** *p* < 0.01, and *** *p* < 0.001.

Finally, we compared F-CM and T-CM activity with functional assays. First, we investigated the efficacy of modulating protease activity in an ex vivo model of osteoarthritis (OA). We used a well-established model where human cartilage explants, challenged with TNFα and IL-1β, exhibit a significant increase in the activity of matrix metalloproteinases (MMPs), a family of matrix-degrading enzymes strongly involved in OA progression [16]. In this context, F-CM and T-CM reduced the protease activity of cytokine-stimulated cartilage explants to the same extent (−93 and −87% with respect to cytokine-stimulated explants, Figure 5a). Secondarily, in order to investigate if the reduction in immunomodulatory factors in T-CM (Figure 1b–i) affects its chemoattractant features, we assessed monocyte migration using a chemotaxis assay with THP-1 cells. Despite the differences in CM composition, no differences between the chemoattractant properties of F-CM and T-CM were detected. Indeed, both cell-free products show significantly higher efficacy than negative control in recruiting THP-1 cells (Figure 5b).

## 4. Discussion

Therapeutics based on cell-derived products are effective tools to address several diseases, such as resolving inflammation in osteoarthritic joints [19] and combating COVID-19 symptoms [20]. They also stimulate restorative mechanisms within the framework of regenerative rehabilitation [21]. However, their inherent variability poses significant production challenges. CM, in particular, relies on a complex mix of proteins, vesicles, and other bioactive molecules for its efficacy. Over the past decade, we have extensively investigated its composition using advanced techniques such as proteomics and lipidomics with mass spectrometry, immunoassay, and Raman spectroscopy [15,22]. These studies have also provided composition-based evidence supporting its potential applications in various clinical contexts, particularly in bone and soft tissue regeneration and immunomodulation, as recently reviewed [23]. Therefore, it is crucial to minimize manipulation and ensure as much standardization as possible during its production [13]. The instability of proteins, lipids, and nucleic acids during freezing and thawing cycles is a significant challenge for preserving biological materials intended for therapeutic use, and their quantification is often employed as a quality control metric [24]. Temperature-induced structural alterations in biomolecules can lead to degradation, aggregation, and loss of function. Proteins are highly sensitive, particularly the ones with complex tertiary and quaternary structures [25]. The formation of ice crystals during freezing causes cryo-concentration of solutes in the sample by segregating them into the unfrozen phase [26]. This increases the concentration of proteins in the unfrozen liquid phase, leading to osmotic stress due to shifts in pH. These changes destabilize protein backbones and disrupt secondary structure interactions, both of which are critical for maintaining protein integrity [27]. During thawing, the uneven exposure of normally buried protein segments to concentrated solutes can result in partial unfolding, potentially leading to misfolding or irreversible aggregation. This phenomenon is particularly frequent at low temperatures, especially in globular proteins, which depend on the confinement of specific segments to maintain stability and functionality [28]. Furthermore, the impairment of stabilizing structures, such as post-translational modifications (PTMs), can exacerbate protein instability. Indeed, PTMs are vital not only for protein localization and mobilization but also for their stabilization. This instability is particularly problematic for bioactive mediators such as growth factors, monoclonal antibodies, and enzymes. Misfolded or aggregated forms of these proteins not only lose their biological activity but may also trigger immunogenic responses [29]. To address these challenges, recent strategies have focused on protein engineering to incorporate stabilizing PTMs into native proteins, thereby enhancing their resilience during storage and processing [30]. Considering that pre-processing freezing is the only difference between samples in this study, the observed reduction in protein content in the T-CM is likely due to a combination of temperature-induced structural alterations and the chemical principles underlying the Bradford assay. In this assay, the dye Coomassie Blue R 250 binds to specific amino acids, primarily arginine, lysine, and histidine, and undergoes a structural shift from its protonated to its unprotonated form (shift in maximum absorbance from 465 nm to 595 nm) [31,32]. This shift in absorbance is what the Bradford assay measures for protein quantification. Structural changes or aggregation in proteins can obscure these amino acid residues, preventing the dye from binding effectively and leading to an underestimation of protein concentration. Given the complex matrix of CM, in terms of protein structures, three-dimensional availability of peptides, and potential reducing agents, protein quantification becomes highly challenging and context dependent. An enzyme’s sensitivity to freezing and thawing cycles, due to its precise three-dimensional conformation critical for catalytic activity, may determine loss of functionality. Freezing disrupts the active site and enzyme–substrate interactions, with cryo-concentration, ice crystal formation, and phase separation, all contributing to enzyme destabilization, partial denaturation, or inactivation [33]. To address the impact of the freezing–thawing step on functional aspects, we evaluated bioactive mediators and assessed potential impairments in anticatabolic and chemotactic properties. Eight factors associated with regenerative and immune-regulatory processes were quantified in both F- and T-CM samples. IL-4, IL-8, CCL2, CCL3, and M-CSF play critical roles in modulating and recruiting immune effectors such as monocytes, neutrophils, and macrophages [34,35,36]. VEGFA and HGF are essential growth factors involved in angiogenesis [37], tissue regeneration, and wound healing [38]. OPG functions as a bone-protective factor, inhibiting osteoclast activation [39]. All these mediators were reduced to varying degrees in T-CM compared to F-CM, likely due to protein instability caused by the freezing–thawing step, particularly for proteins sensitive to extended storage at −80 °C. Despite these reductions, both the anticatabolic and chemotactic properties of F-CM and T-CM remained comparable. Specifically, T-CM retained the ability to inhibit the abnormal MMP activity in cytokine-stimulated cartilage explants and to recruit THP-1 cells in a transwell migration model with no significant differences in efficacy compared to F-CM. These findings are particularly encouraging given the previously discussed drawbacks of freezing and thawing cycles, which are known to negatively impact the activity of individual proteins, enzymes, or compositionally defined samples. Our results suggest that the multifaceted and enriched nature of CM, containing a complex mixture of concentrated bioactive effectors, may confer functional resilience through a “collective” compensatory mechanism that single-molecule therapeutics lack.

In the context of lipid-based biological effectors, such as EVs or liposomal drug delivery systems, freezing and thawing pose additional risks. Ice crystals can physically disrupt lipid bilayers, leading to vesicle content leakage and lipid phase separation, which cause loss of biological factors and encapsulated molecules’ integrity [40]. In therapeutic applications, where EVs are used for their paracrine signaling capabilities—modulating inflammation, promoting tissue regeneration, and delivering regulatory nucleic acids such as miRNAs—freezing significantly reduces their efficacy [41]. Therefore, determining the morphology and concentration of EVs and particles are mandatory in CM characterization. NTA provides particle size and concentration data by tracking Brownian motion of illuminated particles. Although NTA effectively measures these parameters, it cannot reliably discriminate among extracellular vesicles, protein aggregates, or other particles [42,43]. The variation in particles dimensions between F-CM and T-CM suggests partial disruption of larger vesicles during freezing–thawing steps and that the remaining detriments were detected as smaller particles by NTA. This highlights the limitations and biases of relying solely on particle concentration as a quality parameter. Further analysis on vesicles included the cytofluorimetric evaluation of CFSE-stained EVs. In this context, CFSE serves as a convenient stain because only intracellular esterases convert it into its fluorescent form, enabling the identification of intact EVs, while excluding other aggregates [44]. As canonical vesicular markers, we selected the tetraspanins CD9, CD63, and CD81 since they are consistently present on vesicular membranes and are recommended by standard guidelines in EV analysis [8,45]. In this study, TEM analysis was employed as a recommended control to confirm the presence of EVs in the sample. While TEM allowed visualization of vesicles of varying dimensions, it is not a suitable methodology for accurately determining the particle size distribution within a sample that was analyzed by NTA according to approved guidelines [8,45].

The application of Raman spectroscopy to CM quality assessment is in line with the need of high-throughput, reliable, and wide-range analytical techniques. It employs the vibrational analysis of molecular bonds to infer the biochemical composition of a sample [46]. This technique was already applied in previous works and was informative and consistent for CM characterization [47,48]. Indeed, it is also mentioned as an approved methodology in MISEV guidelines [8,45]. In this case, we aimed to assess whether pre-concentration storage at –80 °C altered the final product, and PCA of F-CM and T-CM spectra revealed changes in protein and lipidic composition. In particular, the alterations detected in the P/L ratio in the amide I and amide III ranges suggest that the balance was pushed toward the lipid side in the T-CM. As for the previous results on protein content and EV size distribution, the alterations that happen during freezing and thawing cycles can explain this difference. Until now, the lipid content in the cell secretome has been primarily characterized with a focus on membrane phospholipids [49]. However, lipids also perform essential biological functions beyond their structural roles. They serve as energy substrates and act as signaling mediators. Previously, several lipids were quantified in our CM, including SEA, PGE2, PEA, PGF2, DHA, AA, and EPA [15]. These are part of the bioactive lipid class, which includes molecules involved in key processes such as regulation of cell cycle, senescence, adhesion, migration, inflammation, angiogenesis, and intracellular trafficking [50]. Furthermore, in recent years, several new approaches in drug delivery have begun exploiting lipids to carry functional molecules [51]. The effects of freezing on these lipid structures, however, remain not fully understood. Some best practices to minimize their disruption during freezing include controlled temperature reduction and the use of cryoprotectant agents [52].

In conclusion, freezing is a necessary step for product storage; however, pre-processing freezing of the starting material can alter the downstream manipulation by eliminating some crucial CM effectors. This work underscores the importance of standardized production protocols and transparent quality control measures from pre-processing to final evaluation. Such improvements will enhance the strengths of CM, including extended shelf life, ease of storage, and cost-effective mass production. However, this study specifically focuses on the variations introduced during the freezing and thawing steps in the manufacturing of CM. Expanding the scope to include other variables in the production process would be valuable in determining whether alterations in different processing steps could similarly impact the final product. Additionally, incorporating more functional tests relevant to other therapeutic applications of CM would further strengthen this work. Nonetheless, the key takeaway from this study is the critical importance of scrutinizing every step and manipulation during production, even those that are widely used and endorsed, as they can significantly affect the composition of the product. Future advancements in cell-free therapeutics should integrate all these aspects to facilitate broader applications of CM in immunomodulation and regenerative medicine, such as chronic and acute inflammatory disease, scar and wound healing, and cartilage repair. These insights could inform and refine current guidelines for CM production by emphasizing the need for stringent control and optimization of processing steps, as well as exploring strategies to mitigate freezing-related alterations, such as optimizing cryopreservation protocols or incorporating stabilizing agents to preserve critical bioactive components, without increasing manipulation, toxicity potential, and the overall production’s costs.

## 5. Conclusions

This study highlights the importance of carefully considering each additional step in the production of biotherapeutics, particularly secretomes/conditioned media and EVs. Specifically, freezing the CM at −80 °C prior to further processing caused significant changes in particle composition as well as protein and lipid content compared to the regularly processed CM. This study underscores the importance of carefully evaluating the impact of each step on the overall process.

## Figures and Tables

**Table 1:** ASC donor characteristics.

Sex (*n*)		Surgical Procedure (*n*)			Age (Years)		
*Female*	*Male*	*THR* ^1^	*TKR* ^2^	*AS* ^3^	*Mean*	*Min*	*Max*
4	3	5	1	1	60	35	74

^1^ Total Hip Replacement; ^2^ Total Knee Replacement; ^3^ Aesthetic Surgery.

## Data Availability

The datasets generated for this study will be found in the Zenodo repository at the following link: https://zenodo.org/records/14843382.

## Supplementary Materials

The following supporting information can be downloaded at: https://www.mdpi.com/article/10.3390/biology14020181/s1, Supplementary Figure S1—Representative spectra of different F- and T-CM pairs: *n* = 4 pairs of fresh and thawed CM are represented separately, with black curves for F-CM and red for T-CM.
