# Supplementary figures and images for: Pre-Concentration Freezing Alters the Composition of Mesenchymal Stem/Stromal Cell-Conditioned Medium
Source: Biology (Basel). 2025 Feb 10;14(2):181. doi: 10.3390/biology14020181 (PMC11852129; doi:10.3390/biology14020181)

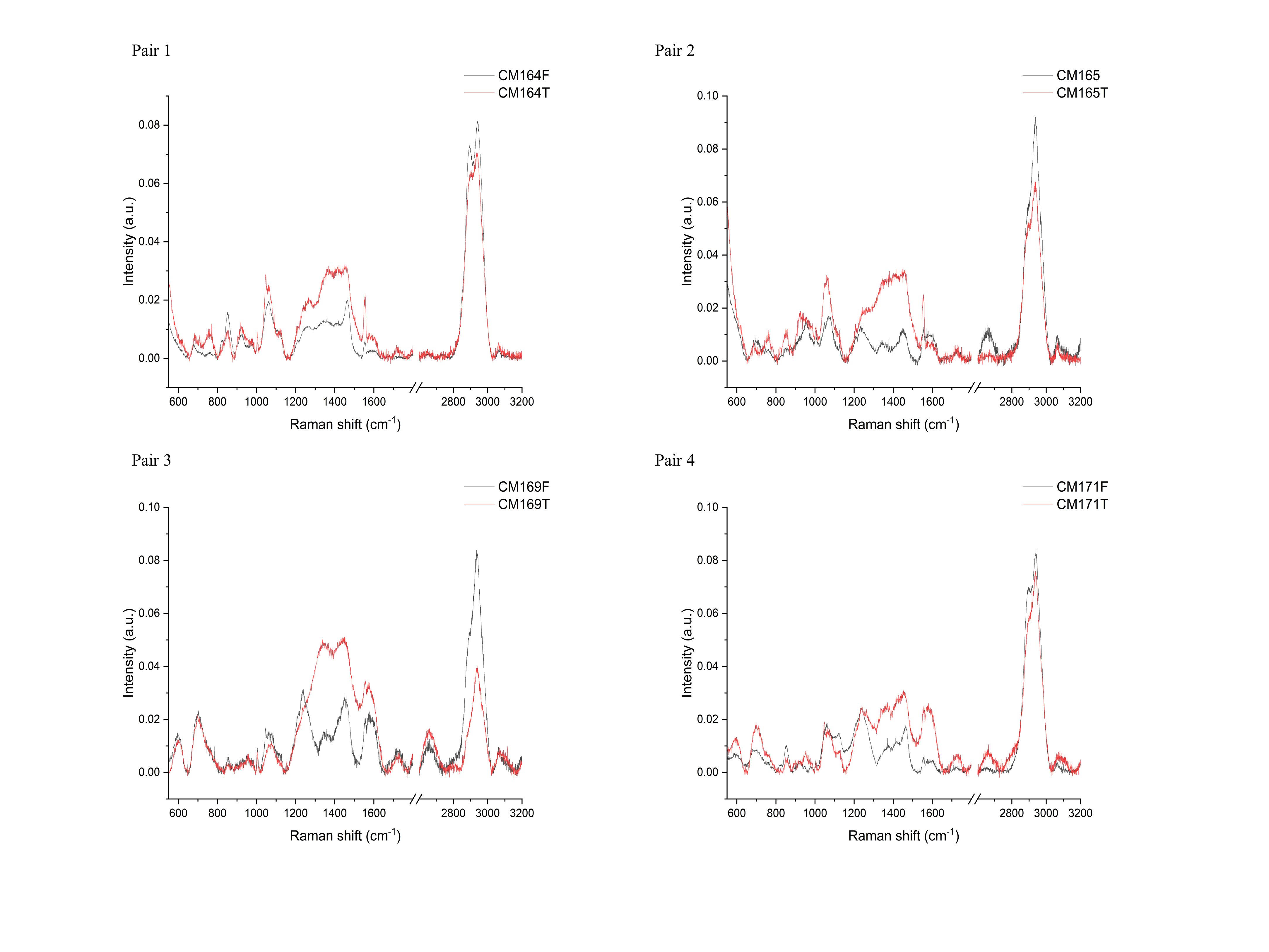

Supplement: Supplementary file 1 [file biology-14-00181-s001.zip › biology-3412175-supplementary.tif]
